# A conflicted tribe under pressure: A qualitative study of negative workplace behaviour in nursing

**DOI:** 10.1111/jan.15491

**Published:** 2022-11-17

**Authors:** Natasha Hawkins, Sarah Yeun‐Sim Jeong, Tony Smith, Jenny Sim

**Affiliations:** ^1^ The School of Nursing and Midwifery The University of Newcastle Callaghan New South Wales Australia; ^2^ Faculty of Medicine and Health, School of Nursing University of Sydney Sydney New South Wales Australia; ^3^ Department of Rural Health The University of Newcastle New South Wales Taree Australia; ^4^ The School of Nursing and Midwifery The University of Newcastle Gosford New South Wales Australia; ^5^ School of Nursing University of Wollongong Wollongong New South Wales Australia; ^6^ Australian Health Services Research Institute (AHSRI) University of Wollongong Wollongong New South Wales Australia

**Keywords:** acute care, bullying, health services research, leadership, nurses, organizational behaviour, workforce issues

## Abstract

**Aim:**

This study explored workplace interactions of Australian nurses in regional acute care hospitals through an examination of nurses' experiences and perceptions of workplace behaviour.

**Design:**

This research is informed by Social Worlds Theory and is the qualitative component of an overarching mixed methods sequential explanatory study.

**Methods:**

Between January and March 2019, data were collected from 13 nursing informants from different occupational levels and roles, who engaged in semi‐structured, in‐depth, face‐to‐face interviews. Data analysis was guided by Straussian grounded theory to identify the core category and subcategories.

**Results:**

Theoretical saturation occurred after 13 interviews. The core category identified is *A conflicted tribe under pressure*, which is comprised of five interrelated subcategories: *Belonging to the tribe*; ‘*It's a living hell*’; *Zero tolerance—*‘*it's a joke*’; *Conflicted priorities*; *Shifting the cultural norm.*

**Conclusion:**

This study provides valuable insight into the nursing social world and the organizational constraints in which nurses work. Although the inclination for an individual to exhibit negative behaviours cannot be dismissed, this behaviour can either be facilitated or impeded by organizational influences.

**Impact:**

By considering the nurses' experiences of negative workplace behaviour and identifying the symptoms of a struggling system, nurse leaders can work to find and implement strategies to mitigate negative behaviour and create respectful workplace behaviours.

**Patient or Public Contribution:**

This study involved registered nurse participants and there was no patient or public contribution.

**Clinical Trial Registration:**

Study registration Australian New Zealand Clinical Trials Registry (Registration No. ACTRN12618002007213; December 14, 2018).

## 1 INTRODUCTION

Conflict related to negative workplace interactions in the nursing profession has deep historical roots and despite extensive research into the problem it continues to persist and has been recognized as a global phenomenon (Hartin, 2021; Minton et al., 2018). Nurses are reported to face a greater risk of exposure to negative workplace behaviours due to the high‐stress environments in which nursing work is situated and the high level of personal involvement nurses have in their work (Shorey & Wong, 2021; Waschgler et al., 2013). A prevalence review indicated that up to 87% of nurses worldwide have experienced negative workplace behaviour over the course of their careers (Bambi et al., 2018) and up to 60% of nurses will leave their first job due to the negative behaviours of their co‐workers (Clarke et al., 2012).

Negative workplace behaviour in nursing has been described as a ‘silent epidemic’ (Murray, 2009, p. 273) due to the acceptance of behaviours as the cultural norm in the profession and victims, therefore, being hesitant to report the behaviour (Hartin et al., 2020; Hawkins et al., 2019). Victims' hesitance to report may be due to a perceived lack of action by management and fear of consequences, such as an escalation of the behaviours in retaliation, and exclusion from work groups (Arnetz et al., 2019; Hartin et al., 2020). Victims of negative workplace behaviour have described feeling unsupported by management to report the behaviours and experienced dissatisfaction and mistrust when the complaints are not acted on (Hallberg & Strandmark, 2006). Over time, this contributes to a reluctance to report and tolerance of negative behaviours (Hawkins et al., 2019). As a result, negative workplace behaviours have become accepted as a ‘rite of passage’ (Birks et al., 2018, p. 48) and an accepted part of nursing socialization (Birks et al., 2018; Moore et al., 2013). Studies indicate that nurses who have previously been subjected to negative workplace behaviour often then treat others in the same way, perpetuating cyclic negative behaviour in the profession (Hawkins et al., 2021a; Lee et al., 2013).

### 1.1 Background

Negative workplace behaviours can be classified into three categories: work‐related bullying (e.g. unmanageable workloads or withholding information); person‐related bullying (e.g. being humiliated or ignored); physically intimidating bullying (e.g. being shouted at or even threats of violence) (Einarsen et al., 2009). Previous studies have found that nurses are more likely to experience work‐related bullying acts, such as unsafe workload allocation (Hawkins et al., 2021a; Palaz, 2013; Yun et al., 2014). Power differentials are also a key aspect of negative behaviour, particularly work‐related negative acts, with the most commonly identified perpetrators being either supervisors or colleagues (Arnetz et al., 2019; Hawkins et al., 2021a; Johnson & Rea, 2009). These behaviours have been found to occur in numerous workplace settings in the nursing profession, ranging from front‐line service provision to senior executive leadership (Edmonson & Zelonka, 2019; Johnson & Rea, 2009).

Individuals exposed to negative behaviours often report psychological distress, poor physical and mental health, decreased job satisfaction, increased absenteeism and intention to leave (Ortega et al., 2011; Trépanier et al., 2016; Wilson, 2016; Wolf et al., 2021). The effects of negative workplace behaviours also place at risk the quality and safety of patients' care, with reported increased incidents of errors, reduced work productivity and avoiding asking for help (Berry et al., 2012; Johnson & Benham‐Hutchins, 2020; Wolf et al., 2021; Wright & Khatri, 2015). Negative workplace behaviour is a barrier to collaborative teamwork and has negative implications for organizations, having been shown to impact staff morale, staff retention and recruitment (Johnson & Benham‐Hutchins, 2020; Ortega et al., 2011; Wolf et al., 2021). For non‐metropolitan health care organizations already faced with difficulties in recruiting and retaining nurses, negative behaviours may impact an organization's employment reputation (Edmonson & Zelonka, 2019; Katrinli et al., 2010; Marufu et al., 2021).

Although there has been extensive research into negative workplace behaviours in the nursing profession, they have been generally in metropolitan settings. Due to the nature of smaller regional locations, health care organizations are often the largest employers in the community (Bushy, 2002; Smith et al., 2019) and nurses working in these locations have limited employment options (Whiteing et al., 2022). Nurses working in regional settings work with limited resources (Smith et al., 2020) in an environment where ‘informal social structures predominate’ (Bushy, 2002, p. 105) and close relationships amongst the community can lead to distortion between professional and personal roles (Bushy, 2002; Mills et al., 2010). Such social intricacies should be considered when examining nurses' experiences of negative workplace behaviour in non‐metropolitan locations.

### 1.2 Theoretical framework

The theoretical framework for this research project is Social World's Theory, which originally emerged from the Chicago School of Sociology (Clarke, 1991). Social world theorists perceive social structure as being shaped and defined by repeated interactions between individuals (Carter & Fuller, 2015), so that society is conceptualized as a mosaic of neighbouring social worlds, which may intersect with each other (Clarke, 1991; Strauss, 1978). Thus, individuals can simultaneously not only belong to but also construct multiple social worlds (Maclean et al., 2021). These social worlds refer to groups where there is ‘a set of common or joint activities or concerns, bound together by a network of communication’ (Kling & Gerson, 1978, p. 26).

Each social world is associated with one primary activity (e.g. delivery of nursing care). There are sites where those activities occur (e.g. in the hospital) and technology applied to carry out the social world's activities (e.g. technical and clinical nursing skills). Unruh (1980) termed the individuals in social worlds as either strangers or tourists or as regulars or insiders based on their ‘social proximity to activities and knowledge vital to the ongoing functioning of a social world’ (Unruh, 1980, p. 280). Each individual in a social world is associated with its activity (e.g. nursing care); however, some individuals are recognized or believe themselves to be more *authentically* of that world. *Authentic* individuals are regarded as most representative of the social world (Strauss, 1982) by those considered to have the ‘power’ to authenticate (Strauss, 1978, p. 123). Power generally resides with those with seniority, authority or longevity in the social world (Strauss, 1978).

Social worlds uphold ideologies about how their work should be completed and in each social world a set of standards of performance are used to evaluate an individual's actions and their degree of *authenticity*, that is, whether an individual belongs in that world (Strauss, 1982). Claims of the *authenticity* of some members and denial of a claim to authenticity of others often lead to conflict in social worlds (Strauss, 1982). The degree of socialization and acceptance felt by individuals who enter a social world impact on how individuals enter and leave (Strauss, 1978). If individuals do not feel fully accepted or are not viewed as being authentic this can lead to conflict and the segmenting of the original social world and the creation of subworlds (Strauss, 1982). Strauss (1982) suggested that the study of conflict and power relationships are crucial aspects of research into social worlds, including the allocation, assigning and depriving of resources (Strauss, 1978).

## 2 THIS STUDY

The overarching research study design is a mixed methods sequential explanatory study with an embedded experimental component. The aim of the overarching study is to investigate the self‐reported exposure to and experiences of negative workplace behaviours of nursing staff and their ways of coping in regional acute care hospitals before and after workshops have been implemented in the organization. The study protocol is described elsewhere (IRRID: PRR1‐10.2196/18643, Hawkins et al., 2021b).

### 2.1 Aim

This article reports on the qualitative strand of the overarching study, which aimed to explore the workplace interactions in the social worlds of nurses working in acute care settings of regional hospitals in New South Wales (NSW), Australia and, thus, enhance the understanding of their experiences and perceptions of negative workplace behaviour. The Consolidated Criteria for Reporting Qualitative Studies (COREQ; Tong et al., 2007) has been applied in reporting this study (Table S1).

### 2.2 Participants and recruitment

The overarching research included 12 medical/surgical wards across four NSW regional acute care hospitals. The hospitals were selected due to being similar in size, with the similar provision of services and case mix. Their co‐location in the same Local Health District meant that all four hospitals were under the same executive leadership and were subject to the same bullying and negative workplace behaviour policies, as well as the same human research ethics governance. The primary author (NH) visited each of the hospitals and the wards to present the study's overall aim and recruitment process. Participants were invited to participate in the quantitative survey component and/or a qualitative, one‐on‐one, in‐depth interview. Recruitment flyers were placed in each ward's tearoom, along with recruitment packages, which contained the participant information statement, the various questionnaires and consent to be contacted for an interview. Study participants who met one of the following criteria were invited:

- New graduate nurses in the first 12 months of practice following completion of a Bachelor of Nursing degree.
- Registered nurses who had been employed for more than 1 year at a minimum of 0.6 full‐time equivalents.
- Nurses in leadership roles, including nurse unit managers, clinical nurse educators and clinical nurse specialists employed at a minimum of 0.6 full‐time equivalents.

For the qualitative strand, the intention was to purposively sample the volunteers to ensure a representative sample of nursing roles and hospital sites. Initially, 46 nurses returned consent forms to participate in an interview. They were all sent a further two follow‐up emails seeking confirmation. A total of 13 informants responded to follow‐up emails, who then became the volunteer sample for the qualitative strand. Data saturation was reached with the 13 interviews, therefore, no further recruitment was undertaken.

### 2.3 Ethical considerations

Ethics approval was obtained from the Hunter New England Local Health District Human Research Ethics Committee (NSW HREC Reference No: HREC/17/HNE/596). All informants were provided with study information on the consent form and were free to decide to participate in the research and to withdraw at any stage without any adverse consequences. Due to the sensitive nature of the research topic, informants had the option to bring a support person with them to the interview and the contact details of the free Employee Assistance Program counselling service were provided. Written consent was sought from informants before audio‐recording the interviews. Data collected were de‐identified to ensure confidentiality.

### 2.4 Data collection

In 2019, semi‐structured, in‐depth interviews were conducted with informants by the primary author at an agreed, private location. The initial interview schedule was informed by a published literature review and by the preliminary results from the quantitative strand of the overarching study (Hawkins et al., 2021a) (Table 1). Data collection and analysis occurred concurrently (Corbin & Strauss, 2015). Although limited to a volunteer sample of 13 informants, at the conclusion of the final two interviews the primary author noted that no new information was obtained (Moser & Korstjens, 2018; Roy et al., 2015) and no new theoretical insights or categories were emerging. Therefore, no further sampling was undertaken (Corbin & Strauss, 2015; Trotter II, 2012).

**TABLE 1:** Interview schedule

Background and demographic questions	Attitude and feelings questions
Approximately how many years have you been a Registered Nurse? Approximately how many years have you worked at your current workplace? What role do you currently have?	How do you feel about how other members of the nursing profession support new graduate nurses? What do you think could be changed to better support new graduate nurses? Do you feel accepted and like you belong as a member of the nursing profession? If so, why? If not, why not? Do you agree with the statement that all nurses are accepted and feel like they belong as a member of the nursing profession? If so, why? If not, why not? What suggestions do you have for improving the workplace culture in the nursing profession?

Knowledge questions	Respectful workplace workshop\questions
What do you know about the NSW Health policy on bullying and incivility? What do you understand bullying to be? Can you give me an example of bullying? What do you understand incivility to be? Can you give me an example of incivility? Is there any difference between incivility vs bullying?	Have you attended the respectful workplace modules 1 and 2? *No*… Have you heard about these workshops? Where did you hear about them? Do you have any intention of attending these workshops in the future? *Yes*… Could you please describe how you felt about the respectful workplace workshops? Do you feel that the workshops were beneficial? If yes, what aspects? If not, why not? What should be done differently? After attending the respectful workplace workshops what changes (if any) did, or will you implement? What changes (if any) have you observed in the wards you have been working?

Opinion and values questions	Experience and behaviour questions
What are your thoughts about bullying and incivility in the nursing profession? What can you tell me about the organizational culture in your current workplace? Do you think that new graduate nurses are more likely to be the victims of bullying and incivility? Why do you think this is/is not the case?	What negative behaviours have you experienced or observed in your workplace? Tell me about how you dealt with negative workplace behaviour? Do you feel that this negative behaviour had any effect on your work? Do you feel like this impacted on patient care you deliver? ○ If yes, tell me about how this impacted on your patient care? What positive behaviours have you experienced or observed in your workplace? Have you ever observed negative workplace behaviour towards new graduate nurses? • If yes, please tell me about the situation • How did you respond? How did they respond?

Final questions
Do you have anything further you would like to say about workplace culture? Do you have any questions for me about this research?

Memos and notes were kept by the researcher during the process (Corbin & Strauss, 2015). The audio‐recorded interviews lasted between 32 and 70 min. All but one interview was audio recorded and transcribed verbatim. One informant chose not to consent to audio recording due to fear of reprisal, but that informant consented to note‐taking by the researcher. Demographic information such as nursing role, years in the current position and total years in nursing were collected during recruitment on the participant consent form and confirmed at the beginning of each interview.

### 2.5 Data analysis

The first two interviews were transcribed by the primary author and the remainder by a commercial transcribing service with which the university had a confidentiality agreement. The primary author reviewed all transcripts, by checking them whilst listening to each audio recording. The transcripts were also sent to all participants to review and verify the contents.

Data analysis was guided by Straussian Grounded Theory (SGT; Corbin & Strauss, 2015; Strauss & Corbin, 1990), which is the method of choice when researching in the Social Worlds Theory framework (Clarke, 1991). Although debated by ‘purist’ grounded theory researchers, there have been numerous examples where theoretical frameworks have been utilized and analysis has been guided by grounded theory methods (Edwards & Jones, 2009; Mitchell Jr., 2014; Rosa, 2010; Sen & Spring, 2011; Vasconcelos, 2005, 2007; Vasconcelos et al., 2012). The combination of social worlds theory as a framework with the analytical tools of grounded theory was combined to uncover the social relationships and behaviours of nurses and construct a new understanding of the phenomenon of the negative workplace behaviours occurring in the nursing social world.

To ensure collaboration and reliability in the data analysis process, the interview transcripts were uploaded into NVivo for all members of the research team to access. To validate the data analysis and interpretation, both the second and third authors (S.Y.‐S.J. and T.S.) reviewed several transcripts, and the findings were discussed with N.H. to reach a consensus. Throughout the data analysis process, the research team met frequently, allowing for comparison and meaningful discussions about how the coding was approached (Harding & Whitehead, 2013; Saldaña & Omasta, 2016).

**FIGURE 1:**
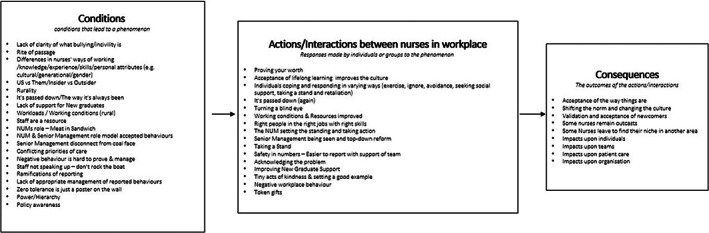
Axial coding using paradigm.

**FIGURE 2:**
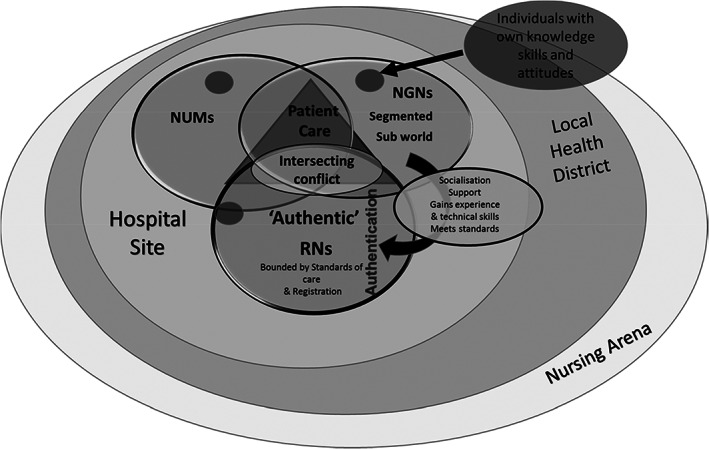
Working diagram of the nursing social world.

Data analysis in SGT is undertaken using a three‐stage approach: open coding; axial coding; and selective coding (Bryant & Charmaz, 2019; Corbin & Strauss, 1990). In the initial stage of open coding, the research team immersed themselves in the transcribed interview data, coding line‐by‐line (Bryant & Charmaz, 2019). The researchers undertook constant comparison, reading and re‐reading transcripts and sorting conceptually similar data into concepts. Memos were also kept and dated during data analysis (Corbin & Strauss, 1990; Harding & Whitehead, 2013; Strauss & Corbin, 1998). The axial coding stage involved the identification of relationships between the concepts (Strauss & Corbin, 1990), utilizing a coding paradigm to assist with analysing, refining and aligning codes (Corbin & Strauss, 1990; Vollstedt & Rezat, 2019). Corbin and Strauss' (2008) coding paradigm consists of three components: conditions, actions/interactions and consequences. The research team ‘put the data back together’ (Corbin & Strauss, 1990, p. 97) by first grouping concepts into those three components. By examining the individual concepts and their interplay, a detailed explanation of the phenomena was developed (Strauss & Corbin, 1998, p. 124; Figure 1). This allowed the researchers to then consider relationships between concepts and link them into broader categories. In the selective coding stage, data categories were refined and integrated to develop a single‐story line (Corbin & Strauss, 1990; Starks & Brown Trinidad, 2007) (Table 2). During that final stage, the research team completed diagramming, as recommended by Corbin and Strauss (1990), as a useful tool for assisting in the integration of categories (Figure 2). A core category and five subcategories were identified that summarize the main ideas of the study.

**TABLE 2:** Overview of categories and subcategories

Core category	Subcategories	Concepts	Coding paradigm
A conflicted tribe under pressure	Belonging to the tribe	Us vs. Them	Condition
		Rurality	Condition
		Rite of passage	Condition
		Power and Hierarchy	Condition
		Ways of Working	Condition
		The way it's always been	Condition
		Proving your worth	Action/Interaction
		Validation and acceptance of newcomers	Consequence
		Remain as outcasts or leave the ward to find their niche	Consequence
	‘It's a living hell’	What is bullying and incivility	Condition
		Negative behaviours	Action/interaction
		Impact on individuals/teams/patient care and Organization	Consequence
		Individuals' way of coping	Action/Interaction
		It's passed down again	Action/Interaction
	Zero Tolerance—‘It's a joke’	Policy awareness	Condition
		Zero tolerance is just a poster on the wall	Condition
		Reporting and its ramifications	Condition
		Do not rock the boat and do not speak up	Condition
		Safety in numbers	Action/Interaction
		People turn a blind eye and it's accepted as the norm	Action/Interaction
		Lack of management of reported behaviours	Condition
		Token gifts	Action/Interaction
		Negative behaviours are hard to prove and manage	Condition
		Staff are a resource	Condition
		Management role model accepted behaviours	Condition
		NUM sets the standard	Action/Interaction
	Conflicted priorities	Working conditions (staffing and workloads)	Condition
		Lack of support for new Graduates	Condition
		Conflicting priorities of care	Condition
		Senior management disconnect	Condition
		Num—meat in sandwich	Condition
	Shifting the cultural norm	Tiny acts of kindness	Action/Interaction
		Shifting the norm	Consequence
		Setting a good example	Action/Interaction
		Acceptance of lifelong learning	Action/Interaction
		Improving new graduate support	Action/Interaction
		Setting the standard and taking a stand	Action/Interaction
		Acknowledgement of problem	Action/Interaction
		Right people right jobs	Action/Interaction
		Management to be seen and need for top‐down reform	Action/Interaction
		Working conditions and resources improved	Action/Interaction

### 2.6 Rigour and Trustworthiness

The research team used a series of techniques to improve credibility, transferability, dependability and confirmability (Lincoln & Guba, 1985). To ensure credibility, the primary author reflected on her own experience as a nurse in a rural setting to capture a true reflection of the social reality of the participants. Throughout the study design, data collection and data analysis, a reflective journal was maintained by the primary author, which outlined reasons for methodological decisions. This assisted the researcher to identify and minimize any researcher bias that may have impacted the data collection, analysis and interpretation. To enhance transferability, the authors have provided a rich description of the research context, informant characteristics and the processes involved in the design, data collection and data analysis stages of this study. This description allows readers to evaluate the applicability of the results to other contexts (Forero et al., 2018; Johnson et al., 2020). An audit trail and memos were kept throughout the analysis to demonstrate the dependability in the decision‐making process (Table S2). To ensure confirmability, the research team also met and debriefed regularly, allowing for peer scrutiny of the analysis and interpretation, thus ensuring that the findings remained true to the informant's accounts of their experience of negative workplace behaviour.

## 3 FINDINGS

A total of 13 informants (4 males and 9 females) participated in this study. They consisted of three New Graduate Nurses (NGNs), one Registered Nurse (RN), six Clinical Nurse Educators (CNEs) and three Nurse Unit Managers (NUMs). They had worked in their current workplace for an average of 10.5 years and had been nursing for an average of 17.4 years. Table 3 gives a detailed overview of informants' characteristics.

**TABLE 3:** List of the informants in this study showing their characteristics

Participant	Gender	Ward type	Site of employment	Years in current workplace	Total years in nursing profession
NUM1	M	Medical	C	24	37
CNE1	F	Medical	C	8	8
NUM2	M	Medical	C	7.5	7.5
CNE2	F	Medical	C	25	25
NGN1	M	Surgical	C	1	1
RN1	M	Medical	B	5	5
CNE3	F	Surgical	B	25	38
CNE4	F	Medical	A	14	37
CNE5	F	Surgical	A	11	34
NGN2	F	Surgical	C	1	1
NGN3	F	Medical	C	1	1
NUM3	F	Medical	D	1	19
CNE6	F	Medical	D	13	13
Mean years				10.5	17.4

Abbreviations: CNE, clinical nurse educator; NGN, new graduate nurse; NUM, nurse unit manager; RN, Registered nurse.

The core category that emerged was *A conflicted tribe under pressure*, which represents the conflict in the nursing profession and the internal and external stressors impacting workplace interactions. That core category is comprised of five subcategories: *Belonging to the tribe*; ‘*It's a living hell*’; *Zero tolerance—*‘*it's a joke*’; *Conflicted priorities*; *Shifting the cultural norm*. Each is described below with relevant examples of informants' quotations.

### 3.1 Belonging to the tribe

This subcategory represents the socialization and validation process of newcomers into the existing ‘tribe’ or social world. Informants described their tribes as having an *us vs. them* (CNE1) attitude, where existing staff were described as territorial and new staff had to prove they were worthy of being included. CNE6 described it as *being like a pack*. It was identified that due to their rurality and, hence, a less transient workforce, long‐term personal relationships and alliances often developed amongst staff, leading to cliques in the workplace. These pre‐existing cliques impacted newcomers' socialization into the tribe, and it was acknowledged by informants that new staff to a ward were more likely to be the victims of negative behaviour, as perpetrators saw them as being vulnerable and weak. The negative workplace behaviour they experienced was viewed as a rite of passage, as described in existing literature (Birks et al., 2018, p. 48) and informants spoke about there being an obvious hierarchy.

Informants also identified barriers to the acceptance of individuals on the ward, such as being part of a casual workforce, being from a different cultural background, gender (being male) and different personal attributes, such as *being a little bit left of the norm* or being *quirky* (CNE3), as well as the level of confidence and the pliability of the individual to fit into the wards culture. The ability to do the job and an individual's way of working also emerged as a major contributing factor to belongingness. Informants identified that nurses who did not cope and could not complete the expected workload were often excluded from the team and faced negative workplace behaviours as a consequence. Time management was identified as being a critical capability. There were, however, different opinions amongst informants as to whether completing the allocated workloads was in fact an achievable task. There was also a discrepancy between informants as to the priority of care for patients, with some indicating that traditionally all showers should and would be completed on a morning shift, whereas others viewed this as an outdated model of care.

Informants identified that these expectations and ways of working were ingrained, and things were done a certain way because *that's the way it was always done* (CNE4). CNE5 described it as like a tribal culture:

We come historically from a background that was a military sort of background. There's a lack of critical thinking and a task orientated style of nursing that does not allow a person the freedom to think critically and to explore other ways of doing things. It regiments and expects a person to conform to a way of doing things because that is how it has always been done. If a person questions that they're maybe stepping out of that tribe.

There were noted consequences for individuals stepping out of that tribe and questioning the way it has always been done. Informants described instances where questioning practice or practising in a different way than *what has always been done* (CNE5) led to bullying and uncivil behaviour. CNE1 suggested *It's not until nurses display practice that doesn't fit within the team that it turns nasty*.

Informants reported having to take action and *put in the hard yards* (CNE3) to achieve acceptance and validation and prove their worth to the team. As a result of their actions, demonstrating to the rest of the team that they could be trusted and accountable for their workload, some participants reported they began to feel accepted and like they belonged in the tribe. That feeling of belonging and validation as an individual was reported to occur when they were being asked for their opinion and when they were approached to help, instead of needing help. NUM1 stated they started to feel really accepted in the profession:

I was being asked for my opinion. If somebody else valued my opinion, they obviously felt that I belonged and I knew what I was talking about and to think that I knew better than they did, I might know something they didn't, that made me feel really like part of the profession then, that I was actually contributing something to the profession.

Other informants reported that they remained on the ward not yet feeling accepted. RN1 described *Well for me it's not the profession that I thought it was going to be. Whether or not—I've not found my niche*.

### 3.2 ‘It's a living hell’

Because negative workplace behaviour can be subjective and based on the victim's personal perception and experiences, it was important to explore the informants' understanding of what constitutes negative workplace behaviour. When informants were asked about their experiences of negative workplace behaviour, every informant identified they had experienced and witnessed negative behaviours whilst working in regional acute care hospitals. All informants in this study, at all occupational levels, believed they had experienced negative workplace behaviours, although they had varied conceptual understandings of negative workplace behaviour. Informants described that bullying behaviour occurred in various forms, which was a higher level than incivility. Three informants (NGN1, NUM3 and CNE6) identified that negative behaviour had to be occurring on a regular basis or be repeated behaviour to be termed bullying. Incivility, on the other hand, was described as a more common, lower‐level behaviours, akin to lack of manners.

The behaviours that informants were exposed to in their workplace included unfair rostering and workloads, being made to work outside of their skill level, isolation and exclusion, information being withheld, being undermined and contradicted in front of the team and being singled out and made to look incompetent. One informant described it as a *living hell* (NUM1) and others shared feelings of embarrassment, fear, sadness, tiredness and feeling physically sick. They describe lacking self‐confidence and the motivation to come to work, feeling burnt out and considering leaving, with new graduates questioning their career choice. NGN1 recalled feeling like ‘I didn't know what I was doing, maybe it wasn't for me, maybe I wasn't cut out for this’. RN1 recalls how he felt as a result of negative workplace behaviours:

It absolutely made me feel terrible. I broke down. I cried because I was just so {long pause}. I think it was a combination of just being so tired and being so worried. I felt saddened and disappointed in the profession.

The behaviours also impacted the informants home life with reports of increased irritability at home, increased alcohol consumption and lack of sleep due to thinking about incidents that had occurred. NUM3 described how the impacts of negative behaviours coupled with personal stressors led to changes in her normal behaviour.

I had a lot going on and this on top of it was just horrible. So yeah, I just ‐ I was probably more cranky at home. But I definitely drank more alcohol. I gained more weight.

Informants also reported that these negative behaviours impacted the team overall, because it affected the ward's culture and working environment. It also led to some nurses avoiding seeking help with patient care, although it was really needed. NGN1 reported that he often avoided asking for help during his graduate year, ‘If it was a minor thing, I just wouldn't worry about it, and I just wouldn't ask any questions’; however, he also conceded that ‘I didn't really know what I was doing. At the time I guess it felt minor’. The negative behaviours also impacted nurses' attitudes and ability to care with many reporting that they were not able to smile at work or even think, which led to a lot more mistakes being made. CNE5 surmised: ‘If you haven't got your needs met, how can you possibly look after someone else?’. Informants indicated that negative behaviours directly put patients at risk as they did not get the care they deserved.

In response to their exposure to negative behaviours, informants reported using various ways of coping, ranging from exercising, avoidance of the person/workplace, keeping work/life separate, giving up and acquiescing to fit in, or formulating a plan to deal with the negative behaviour. Informants also reported seeking support from others including family, mentors and also from Employee Assistance Programs. Although some informants reported taking sick leave or considered leaving their job, several informants reported that due to personal reasons they just had no choice but to just keep going to work. CNE1 reported, ‘I think a lot of nurses are already burnt out, but we just keep going because we've got mortgages’.

It was also evident that retaliation became a mechanism for coping. Informants reported that in response to negative behaviour experienced they began to withhold information, do the bare minimum and refuse to help others if they had been rude or uncivil in the past. This endemic, cyclical nature of negative behaviour was highlighted by NUM1: ‘It's kind of handed down. It's a that's how I got treated, so that's how I'm going to treat you’. This cyclic negative behaviour was cited by informants as a reason that nurses leave the profession and had implications for recruitment. It was acknowledged that good people were leaving the profession and that staff retention and recruitment was an ongoing issue. CNE2 asked, ‘If we bully our staff how are we going to get new staff?’. She suggested that if you are known in the community as having a culture of bullying, people will be less inclined to apply to work there.

### 3.3 Zero tolerance—‘It's a joke’

The informants confessed to minimal knowledge of their organization's bullying policy; nevertheless, all informants recognized that a zero‐tolerance stance was the expected standard. Informants also expressed disillusionment with zero tolerance, with many stating that ‘it's a joke’ and just an ideal, a poster on the wall, a box to tick and never actually enforced. NUM2 asked, ‘It's zero tolerance but I know somebody who's been here for umpteen years, and it's well known that they're a bully, so why is that allowed? If there's zero tolerance, why is that allowed to continue?’

There were many barriers to zero‐tolerance policy enforcement identified by informants, ranging from a lack of reporting to ineffective management. Several informants reported that there was often a lack of confidentiality when reporting, especially in these relatively small rural locations. They considered it brave to speak‐up, due to fear of ramifications and ongoing consequences. They regularly tolerated the behaviours and attempted to ignore it as they did not feel safe and did not want to be seen as obstructive. Informants who had reported behaviours described the consequences, which included further abuse, exclusion from the ward, unfair workload allocations and a decrease in rostered hours. Having colleague support in the ward to report the behaviours empowered informants to engage in the formal reporting process. When the experience was shared instead of an informant feeling like they were the only one being targeted, there was a sense of safety in numbers to stand up and report the behaviours. As NUM3 stated:

I found another person that he was bullying. So, I only reported it because I was encouraged by her to do so and she had also reported it. So, she told me about her experience and she had also been talking to another person that he had been bullying and they had also reported their experience. So, I felt like I wasn't alone and that I potentially might be believed. Because I wasn't sure if I'd be believed.

However, this collective support did not occur in every ward, with informants stating that often people would turn a blind eye, ignoring the problem and leaving informants feeling alone and unsupported. NUM1 described an occasion where he was the victim of negative behaviours in front of others, as follows:

I looked around the room at those who had in private supported my concerns, but they were just sitting there with their heads in their hands. I was left alone and unsupported to cop the heated abuse. I felt awful. On that day no one had my back.

When negative behaviours were reported, informants felt a lack of confidence in management to act. They reported feeling disappointed and disheartened after speaking to management about negative behaviours on multiple occasions with no outcomes or resolution, CNE5 reported: ‘I have been over to management a number of times over the years to various managers and it's just not managed up above’. Informants suggested that senior management had very little accountability, took little action and instead gave token rewards to staff to improve culture, such as barbeques, pizza days and bacon and egg rolls. Informants described those actions as ‘insulting’ (CNE1) and ‘having little effect upon culture’ (CNE2).

Nurse Unit Manager informants also spoke about the difficulties managing negative workplace behaviours from their perspective and, even if staff reported the behaviours, little was often done in response. They acknowledged the huge amount of effort and time that went into the performance management of perpetrators and felt that their workloads did not allow time for this. They referred to a difficult process, plagued by an onus of proof on the victim over a long period of time, multiple strike rules allowing the behaviours to continue and what they perceived as nursing union interference and representation of the perpetrator, making it difficult to actually remove anyone from the ward. NUM3 explained that, due to political reasons and an upcoming state election, one staff member was not performance managed by a NUM ‘Because they were a very unionized staff member, they would bring the unions in. They [NUM] were advised not take it any further’.

Nurse unit manager informants spoke about how, if they did actually bother to undertake the performance management process, it was often disregarded by senior management as it was too much work to address the issues at hand and due to staffing issues, they could not afford to lose anyone. Multiple informants shared the view that negative behaviours were ignored and tolerated because staff were a valuable resource and that often that person and their experience were needed despite their behaviours.

They [*perpetrators*] get away with everything because their clinical skills are excellent. They're a resource, they're an asset, you couldn't possibly lose them despite their particular personality traits. So, in that regard it is a bit frustrating because you know no matter what happens, they will find a way out of it (NGN1).

In another case, NUM1 stated, ‘Realistically I'm not going to put in the work to get someone fired. And let's be honest short of stealing drugs or doing something illegal it is impossible to be sacked in this organisation’.

Informants also reported that negative behaviours were often role modelled by those in leadership positions, with demonstrations of power over those below. The NUMs role in the ward was identified as being crucial in setting the tone and standards of behaviour. For example, NGN2 stated, ‘It's the attitudes and actions of the Nurse Unit Manager that set the standard of what's accepted on the ward’. Informants spoke fondly of NUMs who set a clear standard of expected behaviours and enforced that standard.

Our current NUM I think is the best one that we've had that's sorted things out because she really ‐ from the day that she started, made it clear what she will and won't tolerate on the ward and the values expected. She emphasised that we're a team (CNE6).

One informant (NGN2), however, reported difficulties in her ward as the nurse unit manager displayed negative workplace behaviour, which impacted her management of behaviours of other staff in the clique. That reportedly led to those behaviours being ‘accepted as the norm on that ward as that was the precedent being set from management’ *(NGN2)*.

### 3.4 Conflicted priorities

Informants identified that poor workplace conditions such as short staffing and heavy workloads had negative effects on nurse's workplace interactions. Informants reported increased feelings of stress and defeat as they were unable to complete their allocated workloads. The increasing pressure to complete unachievable workloads reportedly led to irritability, sadness and an inability to help other nurses (such as new graduates) with their workloads. NUM3 described negative workplace behaviours as ‘a symptom of a struggling system’.

Informants suggested that nurses on the ward and senior management had conflicting priorities of care, and informants felt like there was a disconnect between the ‘coal face’ (NUM1) and those making decisions. Senior management was accused of never being seen and hiding in their silos, making decisions based on budgets, tick boxes and key performance indicators rather than seeing patient care as the priority. Informants perceived that senior management lacked empathy and did not appreciate how stressful the work environment was. NUM3 explained:

I think from an executive level and a very high executive level, I don't think they really understand what it's like to work on the floor. Nurses are now working in an environment we've created for them, and I don't think those people understand what they've created. There is no respect for what ward nurses do.

The NUMs also identified that they often felt like the ‘meat in the sandwich’ (NUM1, NUM2, NUM3), caught between senior leadership decisions and expectations and providing ward leadership. The NUMs acknowledged that there was a flow on effect from decisions made above them. NUM3 said:

It's really hard because I have a circle of influence and I can't influence what happens above me and there's so many steps above me that really impact down. I mean when we look at morale and we look at the culture of our unit, I can obviously lead with positivity, I can set examples. But I don't necessarily agree with the things that are pushed on the floor staff with their workloads.

### 3.5 Shifting the cultural norm

Although informants spoke about the occurrence of negative workplace behaviours being embedded in nursing culture, they also spoke about instances of individual tiny acts of kindness and positive workplace practices. Simple things such as pleasant introductions, orientation to the ward environment, knowing where the staff toilet and tearoom were located and someone offering to be the person to help if needed, were identified by informants as enabling newcomers to feel welcome. They described how social events, staff morning teas, holiday celebrations or birthdays with cake all helped build camaraderie and help the team to get to know one other. Some informants suggested that it did not have to be anything big, just simply telling people they are doing a good job, building them up or asking how their shift was going was enough to make a difference in someone's day. Informants identified that by setting a good example and displaying respectful workplace behaviour, they hoped to make a difference. For example, NGN 3 described the acts of kindness that made a difference in her feeling like she belonged:

Certain team leaders used to come round and make sure you were okay, to see if they could help. On my birthday they also made me a cake, so that was really nice. When I had to leave one level for another ward, they all gave me a hug because they didn't want me to go, that was also really nice.

The acceptance of learning as a lifelong process was also viewed as a crucial underpinning to positive workplace culture. Informants spoke about having empathy and insight for new staff coming to a ward and suggested there needed to be improved mentoring, support and workload allocations, especially for NGNs.

Informants identified that by standing up for the victims by shutting down gossip, reporting negative behaviours themselves and empowering victims to stand up to and report negative behaviours they were often able to halt negative behaviour. Informants spoke about instances where they had found the courage to stand up for themselves, ‘prove that they weren't going to be walked over’ (NGN2) and call out the negative behaviour, which had led to improvement in their situation. Aside from the individual actions, informants described organizational actions that could transform the cultural norms of the profession and enhance respectful workplace practices. Informants described that for there to be a shift in the culture of the nursing profession, there needed to first be an acknowledgement of the problem. There were suggestions that the often‐hidden negative behaviours needed to be spoken about more openly amongst the workforce, such as by sharing victims' stories and experiences and educating the future nursing workforce about the issues they may face.

Informants also described how having the right people, in the right jobs and with the right skills in the profession would modify behaviour and culture.

We need those people with the skills and personality to lead by example and manage behaviours and those who are approachable to speak up! We need people who truly have a zero tolerance. It needs be more than just the poster on the wall (NUM1).

Informants felt that the presence of senior management and NUMs in the wards would allow for rapport building and improvement of respectful relationships between management and staff and permit firsthand experience of the poor working conditions. Informants identified that nurse‐to‐patient ratios, more staff and increased resources would improve morale, working conditions and, therefore, improve workplace interactions. NGN1 explained:

I honestly think if we just had more staff and more resources, if we reduced the stress levels, that in itself would reduce instability and bullying drastically. Because if you do that, people are less stressed and people are respecting each other more.

## 4 DISCUSSION

The presence of negative workplace behaviour in nursing is not new (Hartin et al., 2018; Hawkins et al., 2021a, 2022). There has, however, been a shift over the years from the perception that negative behaviours are only attributable to individuals' interpersonal conflict to now recognizing the importance and influence of organizational factors (Hawkins et al., 2021a, 2022; Hutchinson et al., 2008; Johnson, 2015). Strauss et al. (1963), have previously conceptualized the features of a hospital organization such as work groups, rules, hierarchies, policies, organizational goals, ideologies, divisions of labour and career lines in which staff interrelate on a daily basis (Maines & Charlton, 1985; Strauss et al., 1963). These organizational features have implications for the workplace interactions of nurses and were apparent in the experiences shared by informants in this study.

During the interview process, informants described the socialization process of becoming an insider and *Belonging to the tribe*. The Informants spoke about work groups being ‘us vs. them’ and depicted an authentication process of perceived strangers in the nursing social world. Conflict and social world segmentation was evident from the informants' descriptions of individual's attempts to be seen as being worthy to be an insider. They shared their experiences of negative behaviours in their workplace and described the impact on their lives as a *living hell*. There was also a view by informants that the standard of *Zero tolerance*, was a joke and suggested that negative behaviours were often not reported or managed. Various components of *a struggling health care system* such as heavy workloads and a lack of staff and resources compounded by conflicting care priorities between management and front‐line staff were recognized as impacting negatively on workplace interactions. Informants highlighted the need for change at various levels to *shift the cultural norms* of the profession.

The study informants described an obligation by individuals to conform to the rules to be accepted. Achieving acceptance required having a certain standard of knowledge and skills but also having attitudes that aligned with the ward's culture and values. Although the explicit standards of performance for nurses in Australia are enshrined in the Registered Nurse Standards for Practice (Nursing and Midwifery Board of Australia, 2016), it was clear that there were also implicit standards in each ward. Strauss (1982) outlined that every social world has standards of performance along with methods of judging whether the standards are met. Informants identified that insider nurses were the authenticators who judged whether standards of performance had been met and they were responsible for deciding who was authentic and accepted. In this study, there were disagreements in the nursing social world, particularly between younger and older generational nurses about the degree of ‘achievability’ of the implicit social world standards of performance and the priorities of nursing care. This disparity, according to Strauss, indicates social world segmentation (Strauss, 1978). It also raises questions as to how new graduate nurses can ever be ‘practice ready’ (Masso et al., 2022) or ‘hit the ground running’ (Wolff et al., 2010, p. 7). They are educated in the university according to the explicit nursing standards of practice but may graduate having little understanding of the implicit standards of practice and ways of working expected in the nursing social world. The informants in this study outlined that implicit, in‐world standards of performance and ways of working were common areas for conflict to occur. The inability to achieve and complete the workload or work in a different way fueled conflict and negative workplace behaviour. In their observational study of 120 hospital employees, Taylor and Taylor (2017) identified that it was common for negative behaviour and ‘tough‐love’ teaching to occur between colleagues with disparate ways of working. These behaviours were often ‘self‐justified’ (Taylor & Taylor, 2017, p. 3115) by perpetrators as a means of patient advocacy and improving colleague performance.

Informants also highlighted a mismatch between the organizational goals and the ideology of nursing care, which also led to conflict. For the informants, the need for management to meet key performance indicators and budgets was perceived to impact the allocation, assigning and depriving of resources. Deprivation of resources can contribute to excessive workloads and inadequate staffing and, along with a lack of overall support from management, was identified as contributing to negative workplace behaviours. The deprivation of resources is emphasized by Strauss as a ‘power feature’ (Strauss, 1978), and has been reported in previous studies (Hutchinson et al., 2009; Vickers, 2014), where misuse of authority, processes and resources (including human resources) was labelled ‘organizational corruption’ (Hutchinson et al., 2009, p. 217). Despite the acknowledgement of organizational corruption and organizational negative behaviours in the literature of the past 13 years, zero tolerance policies remain targeted towards the management of individual behaviours and appear devoid of any framework for responding to and managing negative organizational behaviours. ‘Organizational corruption’ (Hutchinson et al., 2009, p. 217) and tolerance of negative behaviours increase levels of stress on nurses, which creates the perfect environment for the perpetuation of such behaviours. Those behaviours have persisted throughout the years and have become entrenched in the profession, becoming the cultural norm, as reported elsewhere (Hartin et al., 2018). Unfortunately, where acceptance and tolerance of negative behaviours are the cultural norm, there is a tendency for those behaviours to be underreported (Mckenna et al., 2003). Informants in this study identified that often negative workplace behaviours were accepted as the norm in their organization and, so as not to be seen as being obstructive, they often did not report behaviours. Without first fostering a workplace environment that discourages negative behaviours and establishing a culture that encourages reporting as the norm, the true extent of negative behaviours in an organization will remain hidden, thereby impeding attempts at mitigation.

Informants in this study who had reported negative behaviours expressed disappointment in the lack of action taken by management and viewed zero‐tolerance policies as ineffective due to a lack of enforcement. It has been reported previously that many workplaces react inadequately to negative workplace behaviours, leaving workers disappointed by their inaction (Hallberg & Strandmark, 2006). In relation to social worlds, ‘house rules’ may be viewed as fluid and ‘stretched and negotiated as well as ignored or applied at convenient moments’ (Strauss et al., 1963, p. 130). The shortage of nursing workforce in regional areas was referred to frequently as an excuse as to why a zero‐tolerance stance could not be enforced. Staff were viewed as a valuable resource that organizations could not afford to lose, so negative behaviours were tolerated. Regrettably, by conveniently not adhering to the zero‐tolerance policy, recruitment and retention may be further impacted by the perpetuation of the behaviours, contributing to the ongoing nursing shortage.

Given the influence of organizational factors such as workload and staffing, the impact of the global COVID‐19 pandemic on workplace behaviours since this data was collected warrants further consideration. Nurses have been essential to the healthcare response to COVID‐19 (Fernandez et al., 2020) and many around the world have been infected or died in the line of duty (Turale et al., 2020). During the pandemic, whilst attempting to care for higher acuity patients with increased workloads, nurses have endured reported staffing shortages and lack of resources (Chen, 2020; Manzano García & Ayala Calvo, 2020; Turale et al., 2020). Recent research has quantified the impacts on nurses due to the pandemic, with increased reports of fatigue, stress, burnout and intention to leave the nursing profession (El Ghaziri et al., 2022; Falatah, 2021; Raso et al., 2021). With previous research indicating an association between incivility and burnout (Oyeleye et al., 2013), it is hardly surprising that, in a study of 526 nurses in the United States of America, El Ghaziri et al. (2022) found that 37.4% of nurses experienced increased levels of incivility at work during the COVID‐19 pandemic. In the qualitative component of that study, the informants reported feeling ‘on edge’, ‘short tempered and more stressed than usual’ (El Ghaziri et al., 2022, p. 150). Those informants reported that conflict occurred between nurses in relation to clinical skills, COVID patient assignments and heavy workloads (El Ghaziri et al., 2022). The increased pressure that the COVID‐19 pandemic has placed on an already struggling health care system will continue to have ongoing implications, with experts warning that the true extent of the nursing exodus will not be fully felt until after the end of the pandemic (Falatah, 2021; Raso et al., 2021).

### 4.1 Study limitations

There are some limitations to this study that should be considered when interpreting the results. Firstly, this study was limited to a volunteer, convenience sample of 13 nurses located in one regional area of NSW, Australia. Care should be taken if generalizing the results to other countries and cultures, where there may be differing views of negative workplace behaviour and what is acceptable. Additionally, although this study included only registered nurse informants of varying levels (NGNs, RNs, CNEs and NUMs), the intention was not to underrate the role that other levels of nurses (e.g. endorsed enrolled nurses and assistant nurses) play in the delivery of patient care and workplace interactions. The decision to focus primarily on registered nurse informants' experiences was due to the overarching mixed methods design of the study and time, workload and funding constraints. It is acknowledged that the inclusion of informants from the various levels of nursing roles would provide a more comprehensive picture of the whole nursing social world.

## 5 CONCLUSION

Negative workplace behaviours continue to persist in nursing and remain detrimental to individuals, patients and the nursing profession as a whole. This study has given valuable insight into the nursing social world and the organizational constraints that regional nurses work in on a daily basis. Although the inclination for an individual to exhibit negative workplace behaviours cannot be dismissed, this behaviour can either be facilitated or impeded by organizational influences. The mitigation of negative workplace behaviours requires not only the management of individual behaviours but also the consideration and alleviation of associated organizational causative factors.

To decrease turnover and ensure a strong nursing workforce in the future, it is essential that nursing leaders make it a priority to transform regional nursing work environments to be conducive to respectful behaviours. Organizations should consider the profound ripple effect of under‐resourcing and poor staffing on nurses' workplace interactions. Individual nurses also have a responsibility to demonstrate care and respect for one another and should integrate *tiny acts of kindness* towards others into their daily routines.

## AUTHOR CONTRIBUTIONS

All authors have agreed on the final version and meet at least one of the following criteria (recommended by the ICMJE [http://www.icmje.org/recommendations/]): (1) Substantial contributions to conception and design, acquisition of data or analysis and interpretation of data. (2) Drafting the article or revising it critically for important intellectual content.

## CONFLICT OF INTEREST

The authors declare no conflict of interest.

### PEER REVIEW

The peer review history for this article is available at https://publons.com/publon/10.1111/jan.15491.

## Supporting information


Table S1

Table S2

## Data Availability

Data not available. The data that has been used is confidential.
