# Supplementary material for: A conflicted tribe under pressure: A qualitative study of negative workplace behaviour in nursing
Source: J Adv Nurs. 2022 Nov 17;79(2):711–26. doi: 10.1111/jan.15491 (PMC10100446; doi:10.1111/jan.15491)
Supplement: Supplementary file 1 — Table S1 [file JAN-79-711-s002.docx]

**Supplementary Table 1: COREQ (COnsolidated criteria for REporting Qualitative research) Checklist**

| **Topic** | **Guide Questions/Description** |
| --- | --- |
| **Interviewer/facilitator** | All interviews were conducted by the same member of the study team |
| **Credentials** | The interviewer was a PhD candidate |
| **Occupation** | The interviewer was employed as a part time Nurse Academic |
| **Gender** | The interviewer was female |
| **Experience and training** | The interviewer had successfully completed the qualitative subject component of her PhD studies and conducted a pilot interview under the guidance of a PhD supervisor. |
| **Relationship established** | Interviewees were recruited via the standardised recruitment letter and contacted via email to confirm participation and scheduling of the interview. |
| **Participant knowledge of the interviewer** | Participants were advised of the study aims, requirements and reasons for undertaking the research via the standardised participant information statement. |
| **Interviewer characteristics** | Reasons for undertaking the research were included in the standardised participant information statement and was discussed at the beginning of each interview. |
| **Methodological orientation and Theory** | The qualitative component of this study is underpinned by Social Worlds Theory and was analysed using a Straussian version of Grounded Theory. |
| **Sampling** | Participants were asked to volunteer, resulting in a volunteer sample (n=13) |
| **Method of approach** | Initially potential participants were informed of the study in a face-to-face information session, and information packs including consent for interview were left in the tea rooms of selected wards. After the returning of consent forms, those who have volunteered were contacted via email to schedule the interview. |
| **Sample size** | N = 230 |
| **Non-participation** | Due to the sensitive nature of the research topic, it was expected that some participants would not feel comfortable participating in the interviews, however they were still be able to complete the survey and/or the educational intervention as part of the larger mixed methods study. |
| **Setting of data collection** | Data was collected via face-to-face interviews at a mutually convenient time in private setting. |
| **Presence of non- participants** | Due to the sensitive nature of the topic, participants were given the option to bring along a support person. None of the participants chose to bring along a support person. |
| **Description of sample** | The sample included Nurse unit managers, Registered nurses, Clinical nurse educators, Clinical nurse specialists and new graduate nurses from regional acute care settings. |
| **Interview guide** | The research team developed the semi-structured interview guide, which was pilot tested and further refined prior to interviews. |
| **Repeat interviews** | It was not necessary to undertake any repeat interviews. |
| **Audio/visual recording** | The research team audio recorded the interviews with the participants consent. |
| **Field notes** | The interviewer made field notes after each interview. |
| **Duration** | Each interview took approximately 1hr. |
| **Topic** | **Guide Questions/Description** |
| **Data saturation** | Analysis and data collection occurred concurrently and Interviews until such time that no new insights or theoretical categories emerged. |
| **Transcripts returned** | The research team emailed interview transcripts back to participants for confirmation of validity. |
| **Number of data coders** | Three members of the research team were involved in open coding a sample of transcripts. |
| **Description of the coding tree** | The involvement of three members in the coding process allowed for comparison and meaningful discussions about how the coding was approached and to develop the codebook. Coding discrepancies were resolved through discussion and the list of codes was refined until consensus was reached. An audit trail was established showing the analysis process from raw data in the transcripts through to final selective coding and the emersion of themes. |
| **Derivation of themes** | Themes were developed in stage 2 (axial coding) and stage 3 (selective coding) and involved all members of the research team. |
| **Software** | The research team used NVIVO qualitative software in the initial open coding stage. |
| **Participant checking** | A summary of key findings was shared with participants following data analysis to ensure authenticity and validity. |
| **Quotations presented** | Quotations from multiple participants has been used to present the findings to ensure transparency and trustworthiness. |
| **Data and ﬁndings consistent** | The research team intend on includeding an excerpt from the audit trail to ensure transparency of the data analysis and demonstrate association to the reported findings. |
| **Clarity of major themes** | Headings and sub-headings have been used to promote clarity when presenting the findings. |
| **Clarity of minor themes** | Minor themes and description of diverse cases have also been included in the findings where applicable. |

Developed from: Tong A, Sainsbury P, Craig J. Consolidated criteria for reporting qualitative research (COREQ): a 32-item checklist for interviews and focus groups. *International Journal for Quality in Health Care*. 2007. Volume 19, Number 6: pp. 349 – 35
