# Supplementary material for: A conflicted tribe under pressure: A qualitative study of negative workplace behaviour in nursing
Source: J Adv Nurs. 2022 Nov 17;79(2):711–26. doi: 10.1111/jan.15491 (PMC10100446; doi:10.1111/jan.15491)
Supplement: Supplementary file 2 — Table S2 [file JAN-79-711-s001.docx]

**Supplementary Table 2**: Excerpt from Audit Trail

| **Open Coding – It’s hard to manage** | | | **Axial Coding** | | **Selective coding** |
| --- | --- | --- | --- | --- | --- |
| *Quote* | *Informant* | *Code* | *Paradigm component* | *Concept* | Subcategory |
| “Honestly, **it’s so much work** to sack someone” | NUM1 | It’s hard to manage | Condition | Lack of Management of reported behaviour | Zero Tolerance - It’s a joke |
| “The actual process of dealing with bullying is **a really long process and it’s not easy,** it takes a lot of resilience” | NGN1 |  |  |  |  |
| “I think they just more or less think, well **we're going to push it in the too hard basket or under the mat** and they'll go away” | RN1 |  |  |  |  |
| it's **swept under the rug** because it would be **too much work really to address the issues at hand”** | NGN2 |  |  |  |  |
| “It's **not easy** sometimes to manage and **these people are obviously very difficult to manage”** | NUM3 |  |  |  |  |
| **“The HR process can sometimes be very difficult.** **When you're managing interpersonal skills it's very easy to say, organisational skills, workload management, time management. All those kinds of hard-core things are much easier to educate on and support on and you can really measure in a constructive way, change. But with interpersonal skills it's not as easy to do**. | NUM3 |  |  |  |  |
| I think **they've got to have three lots of fails before it goes to HR for discipline** - **like it's crazy. If you follow that process and you do all the reviews, you're essentially doing it as a manager for that person for about seven months. People are really busy, like who's got time to do that.** **Then you end up spending all your energy on your poor performers and you're not spending your energy on all the great things that are going on in your workplace. So, it's - yeah I don't - it's not so simple.** | NUM3 |  |  |  |  |
| I think that's a **lack of wanting to deal with the issues at hand**. For the analogy of **opening a can of worms**, I think you arise the issues with them and **its they don't want to help because it's going to be too hard.** | NGN2 |  |  |  |  |
